# ^13^C-labelled microdialysis studies of cerebral metabolism in TBI patients^☆^

**DOI:** 10.1016/j.ejps.2013.12.012

**Published:** 2014-06-16

**Authors:** Keri L.H. Carpenter, Ibrahim Jalloh, Clare N. Gallagher, Peter Grice, Duncan J. Howe, Andrew Mason, Ivan Timofeev, Adel Helmy, Michael P. Murphy, David K. Menon, Peter J. Kirkpatrick, T. Adrian Carpenter, Garnette R. Sutherland, John D. Pickard, Peter J. Hutchinson

**Affiliations:** a Division of Neurosurgery, Department of Clinical Neurosciences, University of Cambridge, UK; b Wolfson Brain Imaging Centre, Department of Clinical Neurosciences, University of Cambridge, UK; c Division of Neurosurgery, Department of Clinical Neurosciences, University of Calgary, Canada; d Department of Chemistry, University of Cambridge, UK; e MRC Mitochondrial Biology Unit, Cambridge, UK; f Division of Anaesthesia, Department of Medicine, University of Cambridge, UK

**Keywords:** Brain metabolism, ^13^C-labelling, Microdialysis, NMR, Traumatic brain injury, Human

## Abstract

Human brain chemistry is incompletely understood and better methodologies are needed. Traumatic brain injury (TBI) causes metabolic perturbations, one result of which includes increased brain lactate levels. Attention has largely focussed on glycolysis, whereby glucose is converted to pyruvate and lactate, and is proposed to act as an energy source by feeding into neurons’ tricarboxylic acid (TCA) cycle, generating ATP. Also reportedly upregulated by TBI is the pentose phosphate pathway (PPP) that does not generate ATP but produces various molecules that are putatively neuroprotective, antioxidant and reparative, in addition to lactate among the end products.

We have developed a novel combination of ^13^C-labelled cerebral microdialysis both to deliver ^13^C-labelled substrates into brains of TBI patients and recover the ^13^C-labelled metabolites, with high-resolution ^13^C NMR analysis of the microdialysates. This methodology has enabled us to achieve the first direct demonstration in humans that the brain can utilise lactate via the TCA cycle. We are currently using this methodology to make the first direct comparison of glycolysis and the PPP in human brain.

In this article, we consider the application of ^13^C-labelled cerebral microdialysis for studying brain energy metabolism in patients. We set this methodology within the context of metabolic pathways in the brain, and ^13^C research modalities addressing them.

## Graphical abstract

**Figure:**
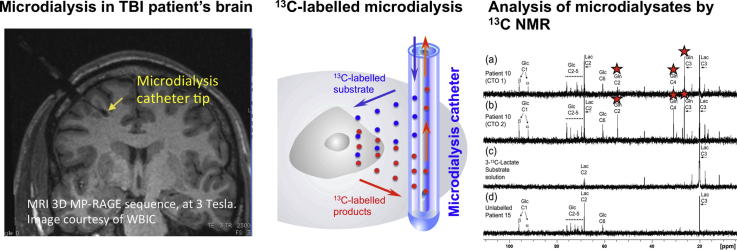

## 1 Introduction

### 1.1 Need for better understanding of energy metabolism in the injured human brain

Energy metabolism in the human brain is incompletely understood, even in the normal uninjured state. Moreover, following traumatic brain injury (TBI), a complex (and variable) sequence of pathological processes arises, in which cerebral energy perturbations appear to play a major role. A well-recognised feature is elevation of brain extracellular lactate and the lactate/pyruvate (L/P) ratio, associated with unfavourable clinical outcome (Timofeev et al., 2011). TBI-induced pathologies evolve over the scale of hours and days, and, despite treatment, may lead to a range of clinical outcomes from good recovery to varying degrees of disability or even death (Kolias et al., 2013). Historically, attention has focussed on ischaemia, leading to a deficiency in supply of oxygen, glucose and other blood-borne nutrients, falling short of the metabolic demands of the injured brain. Much has been learned in neuro-critical care management of patients, so that ischaemia is minimised by adopting modern protocol-driven therapy designed to maintain adequate cerebral perfusion whilst keeping intracranial pressure below a critical threshold. Survival rates, and quality of survival, have thus improved. Despite these advances, there is a growing recognition that, in some circumstances, diffusion barriers (Menon et al., 2004) or increased metabolic demands, e.g. by spreading depression (Parkin et al., 2005), may adversely affect the balance between substrate supply and demand, thus resulting in tissue hypoxia or reduced glucose availability. In other settings, despite seemingly adequate provision of metabolic fuels and oxygen, the injured brain is unable to efficiently utilise these substrates to generate cellular energy. This has sometimes been termed ‘mitochondrial dysfunction’ although the exact basis of this process is not understood. Increased reliance on glycolysis, which produces a low yield of ATP per molecule of glucose, followed by conversion of pyruvate to lactate, is often regarded as a consequence of mitochondrial dysfunction. Also, alternative pathways that consume glucose may become upregulated, such as the pentose phosphate pathway (PPP) that does not generate ATP but is potentially reparative. There is therefore a need to improve our understanding of the fundamental processes of brain energy metabolism, refine our clinical interventions to minimise this energy failure and, ultimately, generate specific strategies to optimise clinical outcome.

Until recently, understanding of energy metabolism has arisen through laboratory investigations, and the concepts applied to humans. Advances in technology are now enabling direct, detailed exploration of metabolism in man. Techniques available include microdialysis, nuclear magnetic resonance (NMR) spectroscopy, mass spectrometry, and *in vivo* magnetic resonance spectroscopy (MRS).

In this article, we consider the application of the novel technique of ^13^C-labelled cerebral microdialysis that we have developed for studying brain energy metabolism in patients (Gallagher et al., 2009). We set this methodology within the context of metabolic pathways in the brain, and ^13^C research modalities addressing them.

### 1.2 Fundamental biochemistry of energy metabolism

**Fig. 1:**
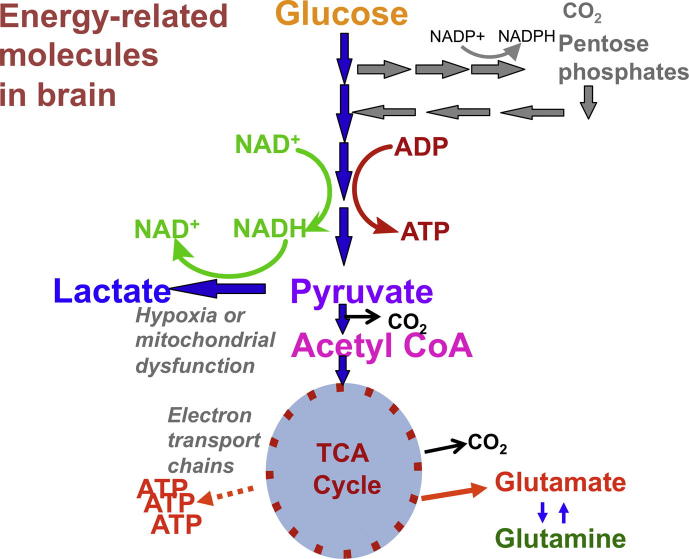
Simplified schematic of major energy pathways in the brain include glycolysis, which takes places in the cytosol and produces pyruvate, which enters mitochondria and is converted into acetyl CoA that enters the TCA cycle. Alternatively, pyruvate can stay in the cytosol and is converted into lactate that is exported out of the cell. The pentose phosphate pathway (PPP) takes place in the cytosol and is an alternative energy pathway that can be up-regulated after injury; it is an important source of NADPH used to produce the reduced form of glutathione (GSH) for preventing oxidative stress.

Energy metabolism is the overall process through which living systems acquire and utilise the energy they need to carry out various functions. Humans, like other chemoorganotrophs, obtain this energy by oxidising organic compounds, notably carbohydrates, lipids and proteins. This energy is coupled to endergonic reactions resulting in the synthesis of high-energy phosphate compounds, specifically adenosine triphosphate (ATP). The primary organic compound utilised by humans is glucose. ATP is generated by a sequence of three well-recognised processes (Fig. 1), as follows:

(a) *Glycolysis:* This is the linear pathway (also termed Embden–Meyerhof–Parnas pathway, or the Embden–Meyerhof pathway) by which glucose is converted (via several intermediates) to pyruvate, thereby generating 2 molecules of ATP per molecule of glucose. Glycolysis does not involve molecular oxygen. After glycolysis, under aerobic conditions, pyruvate is converted to acetyl CoA and enters the tricarboxylic acid (TCA) cycle (see (b), below). Under anaerobic conditions, and/or if the mitochondria are dysfunctional, pyruvate is converted by the enzyme lactate dehydrogenase to lactate, while converting nicotinamide adenine dinucleotide (NADH) to NAD^+^. The L/P ratio can therefore be used as an indicator of the comparative degree of “aerobic” vs. “anaerobic” metabolism. One of the intermediates in glycolysis, glucose-6-phosphate, can also be metabolised via the PPP initially generating nicotinamide adenine dinucleotide phosphate (NADPH) and ribulose-5-phosphate that undergo further reactions (see below). Both glycolysis and the PPP occur in the cytosol.
(b) *Tricarboxylic acid (TCA) cycle:* This cyclical pathway (also termed citric acid cycle or Krebs cycle) comprises a series of 8 reactions that oxidise the acetyl group of acetyl-coenzyme A (acetyl-CoA) producing 3 molecules of NADH, one molecule of flavin adenine dinucleotide (FADH_2_), and one molecule of guanosine triphosphate (GTP). The TCA cycle takes place in mitochondria.
(c) *Oxidative phosphorylation:* This is a process occurring in mitochondria through which NADH and FADH_2_ from the TCA cycle are oxidised by oxygen in the electron transport chain resulting in the synthesis of further molecules of ATP. Also, the succinate to fumarate step of the TCA cycle takes place directly on part of the electron transport chain (Complex 2) thereby promoting ATP synthesis.

Overall, one molecule of glucose, in the presence of oxygen, processed via this three-stage pathway (glycolysis, TCA cycle and oxidative phosphorylation), yields a net production of 36 molecules of ATP.

### 1.3 Brain energy metabolism

**Fig. 2:**
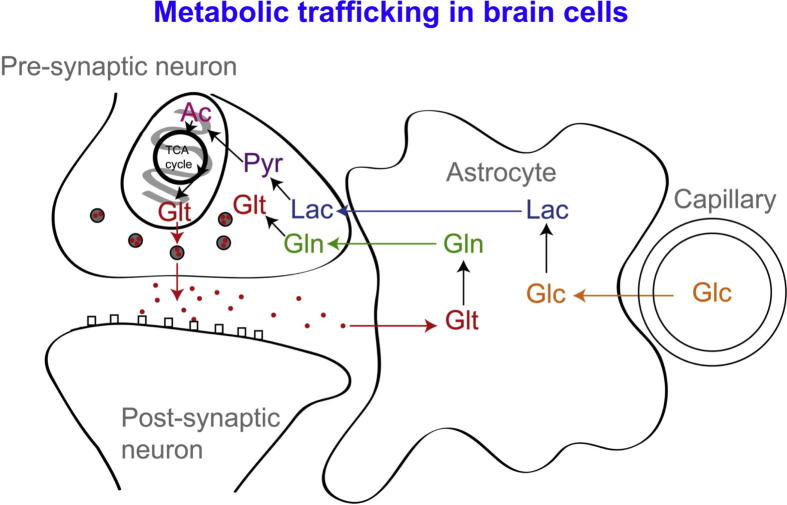
Glucose (Glc) from the vasculature is metabolised to lactate (Lac) in astrocytes, exported into the extra-cellular fluid, taken up by neurons and processed (via pyruvate (Pyr) and acetate (Ac)) by the TCA cycle. This spins off glutamate (Glt), which is released and then taken up by astrocytes, which convert it to glutamine (Gln), which is released into the extra-cellular fluid and taken up by neurons, which re-convert it to glutamate (Gallagher et al., 2009). Figure originally published in Brain (Gallagher et al., 2009).

**Fig. 3:**
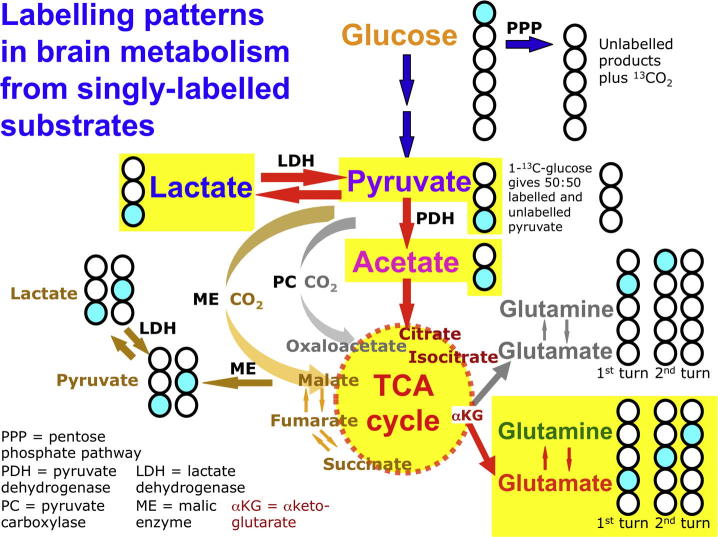
Some examples of ^13^C-labelling patterns in brain metabolism, based on results with singly labelled substrates (Gallagher et al., 2009; Tyson et al., 2003). Turquoise fills indicate ^13^C atoms. Yellow highlight indicates the metabolic pathway by which ^13^C-labelled lactate is processed via the TCA cycle, emerging as ^13^C-labelled glutamate or ^13^C-labelled glutamine that can be recovered in brain microdialysates. (For interpretation of the references to colour in this figure legend, the reader is referred to the web version of this article.)

**Fig. 4:**
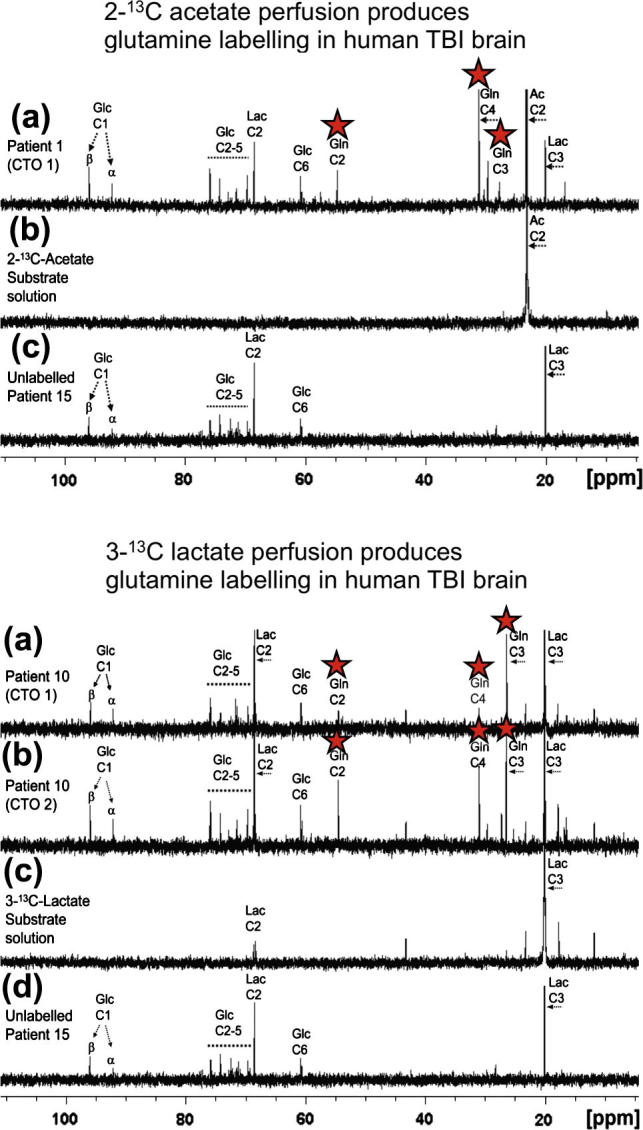
*Upper panel:* (a) Example of ^13^C NMR spectrum of brain microdialysate from a TBI patient, who received perfusion with 2-^13^C acetate (4 mM) by a microdialysis catheter via a craniotomy (CTO); red stars indicate ^13^C signals for glutamine C4, C3 and C2 indicating metabolism via TCA cycle. (b) ^13^C NMR spectrum of the 2-^13^C acetate substrate solution prior to perfusing. (c) ^13^C NMR spectrum of brain microdialysate from an unlabelled patient whose microdialysis catheter was perfused with plain perfusion fluid without labelled substrate. *Lower panel:* (a) and (b) Examples of ^13^C NMR spectra of brain microdialysates from a TBI patient, who received perfusion with 3-^13^C lactate (4 mM) by microdialysis catheters via a craniotomy (CTO); red stars indicate ^13^C signals for glutamine C4, C3 and C2 indicating metabolism via TCA cycle. (c) ^13^C NMR spectrum of the 3-^13^C lactate substrate solution prior to perfusing. (d) ^13^C NMR spectrum of brain microdialysate from an unlabelled patient (as in *Upper panel* (c)). *Abbreviations:* see Fig. 2 legend. For further details, see (Gallagher et al., 2009). Figures originally published in Brain (Gallagher et al., 2009).

While glucose is recognised as the primary substrate in most organs, including muscle, recent evidence suggests that the situation in the brain is considerably more complex, particularly in relation to the main cell types. The traditional view is that both glia and neurones metabolise glucose as the preferred substrate via glycolysis to pyruvate, which is converted to acetyl CoA and enters the TCA cycle resulting in the generation of ATP by oxidative phosphorylation. Recent evidence suggests, however, that the neurones may utilise lactate (classically perceived as a waste product) as an energy substrate. Fig. 2 outlines a scheme of metabolic trafficking between astrocytes and neurons. This theory, later termed the astrocyte-neuron lactate shuttle (ANLS) hypothesis, was originally proposed (Pellerin and Magistretti 1994) as a result of *in vitro* studies and has been later supported by studies in animals e.g. (Tyson et al., 2003). Moreover, our recent microdialysis studies using ^13^C-labelling have demonstrated that the injured human brain can metabolise lactate via the TCA cycle (Gallagher et al., 2009) (Figs. 3 and 4).

Cerebral metabolism following injury appears to differ from that of the normal brain, although the full extent and nature of these changes are poorly understood, especially in man. The best-known metabolic characteristic of injured brain is a high L/P ratio (>25 is interpreted as ischaemia or mitochondrial dysfunction) (Bellander et al., 2004; Hillered et al., 2006; Hillered et al., 2005; Persson and Hillered 1992; Persson et al., 1996). Our study of 233 TBI patients demonstrated that microdialysate L/P ratio >25 predicted unfavourable outcome in a multivariate analysis in addition to previously known predictors of outcome (Timofeev et al., 2011).

Low extracellular lactate levels, associated with better outcomes (Timofeev et al., 2011), might be because astrocytic glycolysis-derived lactate is being efficiently taken up by neurons and utilised via the TCA cycle (Gallagher et al., 2009). Conversely, high extracellular lactate may result if neurons are too damaged to efficiently utilise the lactate being produced by astrocytes, i.e. uncoupling of neuronal and glial metabolism.

### 1.4 Alternative pathways

#### 1.4.1 Pentose phosphate pathway

**Fig. 5:**
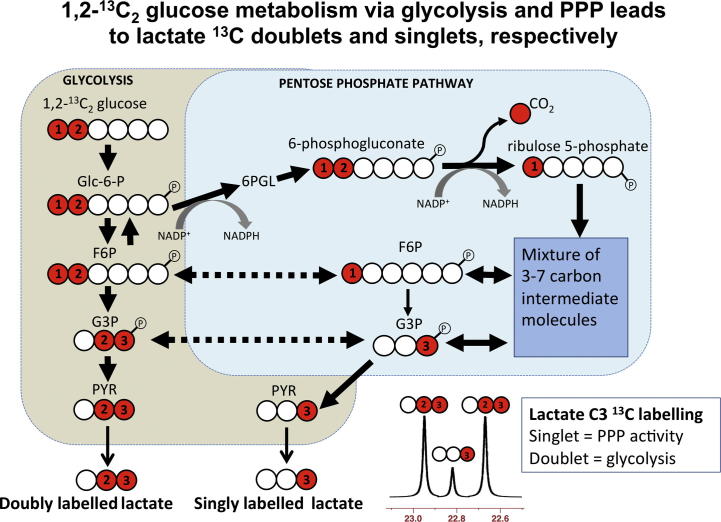
Simplified schematic of steps in glycolysis and the pentose phosphate pathway (PPP), showing ^13^C labelling patterns resulting from 1,2-^13^C_2_ glucose substrate. Red fills indicate ^13^C atoms. *Abbreviations:* Glc-6-P, glucose-6-phosphate; 6PGL, 6-phosphogluconolactone; F6P, fructose-6-phosphate; G3P, glyceraldehyde-3-phosphate; PYR, pyruvate.

As well as the fundamental pathways outlined in Section 1.2, other pathways may also be relevant, for example the PPP (Fig. 5), also termed the hexose monophosphate shunt, a complex detour (hence the name “shunt”) that bypasses some of the steps of glycolysis. Glucose enters the cell, and becomes phosphorylated to give glucose-6-phosphate as in glycolysis, but then in the PPP it is diverted through a different set of enzymatic reactions. Glucose-6-phosphate is oxidised to 6-phosphogluconolactone by glucose-6-phosphate dehydrogenase, the rate-limiting step of the PPP. NADP^+^ is a cofactor, and NADPH is produced. Subsequently 6-phosphogluconolactone is converted to 6-phosphogluconate, and then C1 of the 6-carbon sugar skeleton is lost as CO_2_, together with conversion of NADP^+^ to NADPH, and formation of ribulose-5-phosphate (5-carbon sugar, a pentose). The aforementioned steps are termed the oxidative phase of the PPP. The subsequent steps are termed the non-oxidative phase of the PPP, in which the 5-carbon skeleton undergoes various transformations and recombinations generating intermediate molecules with 3–7 carbon atoms. The 5-carbon (pentose) sugar ribose-5-phosphate can be utilised in nucleic acid synthesis, providing reparative building blocks for the injured tissue, while the 4-carbon sugar erythrose-4-phosphate can be utilised in the synthesis of aromatic amino acids. The NADPH generated by the oxidative phase of the PPP provides reducing equivalents for the synthesis of fatty acids (e.g. for repairing injured tissue) and for the formation of the reduced form of glutathione (GSH), an essential co-factor for glutathione peroxidases (GPx), a family of enzymes that remove hydroperoxides, thereby combating oxidative stress. While the activity of the PPP thus appears potentially reparative, it neither consumes nor generates ATP. The exit-points of the PPP involve the re-entry of products fructose-6-phosphate and glyceraldehyde-3-phosphate (these can alternatively be recycled back into the PPP to generate more NADPH) into the main stream of glycolysis, producing pyruvate, which can enter mitochondria, form acetate and enter the TCA cycle, or pyruvate can be converted to lactate in the cytosol and exit into the extracellular fluid.

Overall, 3 molecules of glucose-6-phosphate (6-carbon sugar) that enter the PPP lose 3 molecules of CO_2_ while converting 6 molecules of NADP^+^ to 6 molecules of NADPH, and producing 3 molecules of ribulose-5-phosphate (5-carbon sugar) which, via several intermediates (3–7 carbons), result in 2 molecules of fructose-6-phosphate (6-carbon sugar) and 1 molecule of glyceraldehyde-3-phosphate (3-carbon molecule). However the stoichiometry is not necessarily so straightforward, as some of the 5-carbon and 4-carbon sugar intermediates can be taken out of the PPP by conversion to nucleic acids and amino acids respectively, and some of the fructose-6-phosphate (6-carbon sugar) and glyceraldehyde-3-phosphate (3-carbon molecule) that are PPP products can be recycled back into the PPP, potentially generating more NADPH, as mentioned above.

Pre-clinical evidence from ^13^C-labelling and NMR suggests that the utilisation of glucose following injury differs from normal, with an increased flux through the PPP, which is upregulated by cellular stress, demonstrated in animals, e.g. brain injury (controlled cortical impact or fluid percussion injury) with brain tissue extracts analysed *ex vivo* using ^13^C NMR (Bartnik et al., 2007; Bartnik et al., 2005), or hypothermia with brain tissue extracts analysed *ex vivo* by ^1^H-observe [^13^C-edited] spin-echo difference NMR (Kaibara et al., 1999). The idea of an increased PPP flux was supported by the results of an indirect measure in a small study of TBI patients (*n* = 6) vs. healthy controls (*n* = 6), using arterio-jugular venous difference (AJVD) with 1,2-^13^C_2_ glucose (intravenous infusion) as the substrate with gas chromatography–mass spectrometry (GC–MS) used for analysing the products including ^13^C-labelled lactate in arterial and jugular venous blood (Dusick et al., 2007). However, the AJVD methodology could only produce relevant ^13^C results in periods when the brain was showing net export of lactate, as opposed to net import, which can happen in some periods (Dusick et al., 2007; Jalloh et al., 2013), and the findings of Dusick et al. have yet to be confirmed in human brain directly. We are currently using 1,2-^13^C_2_ glucose substrate perfused via cerebral microdialysis catheters in TBI patients, with analysis of lactate ^13^C labelling in microdialysates, using ^13^C NMR (see Section 3.1).

Using 1,2-^13^C_2_ glucose as the substrate, the products downstream of the PPP include lactate singly labelled at the C3 position, showing as a singlet in the ^13^C NMR spectrum (Fig 5). Lactate produced via glycolysis (without going through the PPP) is doubly labelled with ^13^C at both the C3 and C2 positions of lactate within the same molecule, showing as a doublet for C3 (Fig. 5), and a doublet for C2. The ratio of C3 singlet to C3 doublet signal intensities (peak area ratios) can be used as a measure of lactate production by PPP vs. glycolysis. Bartnik and colleagues used this approach in pre-clinical studies of brain injury in rats (Bartnik et al., 2007; Bartnik et al., 2005). Brain tissue extracts from the control rats (uninjured, sham-operated) in these studies showed lactate C3 doublet indicative of glycolysis, while C3 singlet was undetectable, i.e. no detectable lactate via the PPP (Bartnik et al., 2007; Bartnik et al., 2005). In the same studies, for brain tissue extracts from the brain-injured rats (controlled cortical impact or fluid percussion injury), Bartnik and colleagues reported detectable lactate C3 singlet and C3 doublet, with up to 13% PPP which they calculated using the lactate C3 singlet/doublet ratio (Bartnik et al., 2007; Bartnik et al., 2005). Endogenous lactate, which naturally contains 1.1% ^13^C at each position, also gives a singlet for each carbon. Therefore, unless the endogenous lactate signal is negligible, the lactate singlet C3 signal needs correcting to subtract any background contribution from natural abundance, before calculating its ratio to the C3 doublet. Endogenous extracellular lactate concentrations are usually quite high (e.g. 2–4 mM) in brain microdialysates of TBI patients (Timofeev et al., 2011), and are readily detectable, even in TBI patients without ^13^C labelling, by ^13^C NMR on the Bruker Avance 500 MHz spectrometer with a cryoprobe (Gallagher et al., 2009) (see Fig. 4). Therefore in our current ongoing lactate labelling studies using 1,2-^13^C_2_ glucose as the substrate, we are making allowance for background in evaluating the results. For further considerations about ^13^C natural abundance background, see Section 4.3.

A model has been developed by Lee and colleagues using 1,2-^13^C_2_ glucose as the substrate, whereby labelling patterns produced in lactate are used to calculate the fraction of glucose metabolic flux that occurs via the pentose cycle (PC), as defined (Lee et al., 1998). Some of assumptions of the model stated by Lee and colleagues include that glucose is mostly metabolised by either the Embden Meyerhof or the pentose pathway, the pentose phosphate produced is recycled to hexose phosphate, and net loss of pentose phosphate to nucleic acid synthesis is relatively small. While a full discussion of the model and all of its assumptions and limitations is outside the scope of the present article for reasons of length, some of the model’s key features are as follows. The initial 1,2-^13^C_2_ glucose study was *in vitro* in HepG2 (hepatoma) cells (Lee et al., 1998). This used GC–MS to measure isotopomer yields of lactate molecules, i.e. the abundances (expressed as molar fraction) of ions with no ^13^C (*m*_0_), one ^13^C (*m*_1_) and two ^13^C (*m*_2_), after correcting for natural abundance of ^13^C (see Section 4.3); note that *m*_0_ + *m*_1_ + *m*_2_ = 1. The formula PC = (*m*_1_/*m*_2_)/[3 + (*m*_1_/*m*_2_)] was derived. This model was later applied *in vivo* in TBI patients, with intravenous infusion of 1,2-^13^C_2_ glucose substrate and *ex vivo* GC–MS analysis of lactate labelling in arterial and jugular venous blood (Dusick et al., 2007); see above. GC–MS reveals how many ^13^C atoms are present in the molecule, but does not distinguish the intra-molecular positions of these ^13^C atoms. Lactate derived from 1,2-^13^C_2_ glucose via glycolysis shows m_2_ labelling revealed by GC–MS, due to ^13^C at both C2 and C3 of lactate adjacent together within the same molecule, as revealed by NMR (see above). Singly labelled lactate derived from 1,2-^13^C_2_ glucose via the PPP shows m_1_ labelling revealed by GC–MS. Primarily this single ^13^C appears at the C3 position of lactate, although recycling within the PPP may result in ^13^C also appearing as single labelling at C2, and as single labelling at C1, all of which would show as lactate m_1_ labelling on GC–MS, without distinguishing between the positions of the ^13^C (Lee et al., 1998). Another potential process whereby lactate single labelling may appear “scrambled” is if 3-^13^C pyruvate interacts with the TCA cycle via the action of malic enzyme (ME), producing lactate with a mixture of single labelling at C3 or C2 (Fig. 3).

The implications of the PPP for energy generation are still not fully understood. The PPP may be regarded as sacrificing some of the carbon atoms from the cells’ supply of glucose (which could otherwise have been used for ATP generation), for the sake of generating more reducing power (NADPH) and the ability to protect, repair or build cells. While the PPP appears upregulated by injury, it also appears up-regulated in cancer (Riganti et al., 2012). Glycolysis also appears upregulated in cancer, a phenomenon termed the Warburg effect (Warburg 1956). Tumours are often highly glycolytic producing large amounts of lactate regardless of the presence of oxygen (Bensinger and Christofk 2012; Warburg 1956). Both the PPP and the Warburg effect may represent potential therapeutic targets. Unlabelled cerebral microdialysis has been used to study brain chemistry in glioma patients (Marcus et al., 2010), and in future such studies may be extended by use of ^13^C-labelled microdialysis.

#### 1.4.2 Anaplerosis

While the well-recognised main entry point into the TCA cycle is where acetyl-CoA joins with oxaloacetate to form citrate, additional entry points exist (Owen et al., 2002), termed anaplerotic reactions (meaning “filling up” or “replenishing”). For example, while the main pathway for pyruvate involves the enzyme pyruvate dehydrogenase (PDH), which converts pyruvate to acetyl CoA that enters the TCA cycle, an anaplerotic pathway involves the reaction of pyruvate with CO_2_ by the action of pyruvate carboxylase (PC) producing oxaloacetate, which is a TCA cycle intermediate (Fig. 3). Anaplerosis is necessary because as well as the TCA cycle generating ATP via mitochondrial oxidative phosphorylation, its intermediates are also sources of amino acids and other molecules for the cell. A notable exit point is at alpha-ketoglutarate, a TCA cycle intermediate that can be converted to glutamate, and thence to glutamine (Fig. 3). The TCA cycle thus needs replenishment, provided by anaplerosis. Fig. 3 illustrates that ^13^C-labelling patterns in the emerging glutamate and glutamine can differentiate between TCA cycle anaplerosis (grey arrows), with pyruvate entry via PC activity, and mainstream TCA cycle (red arrows), with pyruvate entry via PDH and acetyl CoA (Tyson et al., 2003).

## 2 Microdialysis as a tool for monitoring cerebral metabolism

### 2.1 Cerebral microdialysis in multimodal monitoring

**Fig. 6:**
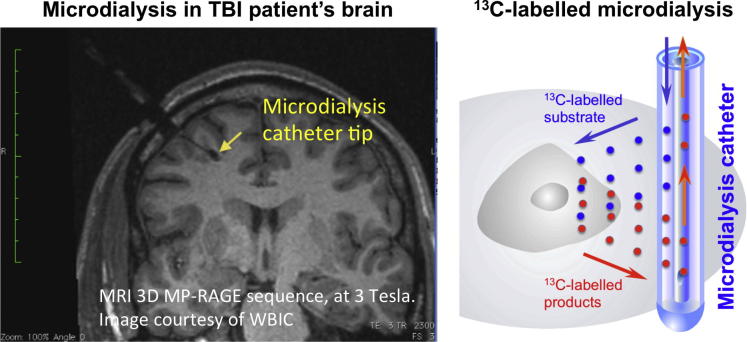
*Left panel:* Magnetic resonance imaging (MRI) scan, using 3D MP-RAGE sequence, at 3 Tesla, showing the brain of a TBI patient with a microdialysis catheter (via a cranial access device) in position, the catheter tip indicated by an arrow (yellow). Scale bar (green) shows centimetre divisions. Image is courtesy of the Wolfson Brain Imaging Centre, Dept. of Clinical Neurosciences, University of Cambridge. *Right panel:* Schematic of ^13^C-labelled microdialysis. (For interpretation of the references to colour in this figure legend, the reader is referred to the web version of this article.)

Monitoring of severe TBI patients in neuro-critical care may include intracranial pressure, brain tissue oxygen and extracellular chemistry using microdialysis. Clinical microdialysis catheters (M Dialysis AB, Stockholm, Sweden) are often inserted through a burr-hole via a cranial access device e.g. a triple-lumen “bolt” (Technicam, Newton Abbot, UK), which allows up to 2 other probes (e.g. for intracranial pressure and brain tissue oxygen monitoring) to be inserted alongside the microdialysis catheter into brain. Microdialysis catheters can also be inserted via craniotomy, without a cranial access device. While insertion via a cranial access device orients the catheter perpendicularly into the brain, the craniotomy situation provides the option of orienting the microdialysis catheter at a shallow angle (i.e. tangentially), to target grey matter (Gallagher et al., 2009). Grey matter can also be contacted by a perpendicularly oriented catheter deeper within the brain, because of the folded nature of the cortex, although this is more difficult to target. The position of the catheter after insertion can be verified using computed tomography (by virtue of the catheter’s gold tip), or magnetic resonance imaging (MRI) (Fig. 6).

Clinical microdialysis catheters possess a semi-permeable membrane (nominal molecular weight cutoff either 20 or 100 kDa) that is continuously perfused with fluid, allowing molecules to diffuse across the membrane, to and from the brain’s extracellular space. For routine clinical monitoring, the catheter is perfused with a physiological salt solution e.g. CNS perfusion fluid (M Dialysis AB, Stockholm, Sweden) and the returning fluid (microdialysate) is analysed at the bedside utilising automated enzymatic colorimetric assays (CMA600 or ISCUS microdialysis analyzer, M Dialysis AB) to measure endogenous glucose, lactate, pyruvate, glutamate and glycerol. Thus, microdialysis has been used to monitor glucose delivery and the balance between aerobic and anaerobic metabolism using the L/P ratio (Timofeev et al., 2011). For our ^13^C labelling studies, the ^13^C labelled substrate (manufactured by Cambridge Isotope Laboratories Inc., Tewkesbury/Andover, MA) is formulated at an appropriate concentration in CNS perfusion fluid, in a hospital pharmacy manufacturing unit, with tests for conformity to standard pharmaceutical regulations for purity, sterility, and freedom from pyrogens. The ^13^C labelled substrate formulation is then analysed by high resolution ^13^C NMR to ensure that there are no impurities that would compromise the studies. The formulated ^13^C labelled substrate is then pumped through the microdialysis catheter in the usual way, and microdialysates collected for analysis (Gallagher et al., 2009).

### 2.2 Cerebral microdialysis for conveying molecules to and from the extracellular space

As diffusion across the microdialysis membrane is bi-directional, microdialysis can deliver molecules (“retrodialysis” e.g. ^13^C-labelled substrates), thereby micro-dosing a region of interest around the catheter tip, whilst simultaneously collecting the downstream products in the emerging microdialysate, for analysis in the laboratory (see Fig. 6 for schematic). In this way, we showed that infusion of 3-^13^C-lactate or 2-^13^C acetate into the brains of TBI patients via the microdialysis catheter produced ^13^C signals for glutamine C4, C3 and C2 in the emerging microdialysates, indicating TCA cycle operation followed by conversion of glutamate to glutamine (Gallagher et al., 2009) (Figs. 3 and 4). Microdialysis can thus be used to probe the immediate microenvironment around the catheter by adding chosen ^13^C-labelled metabolic substrates that enter the biochemical pathway at different steps, allowing relevant stages to be investigated. The extent of the region addressed by the microdialysis catheter is not precisely known. In a combined FDG-PET and cerebral microdialysis study (without ^13^C-labelling) in TBI patients, the cerebral metabolic rate of glucose measurements in a 2 cm region of interest around the microdialysis catheter tip correlated significantly with endogenous lactate and pyruvate concentrations in the microdialysates (Hutchinson et al., 2009).

## 3 Nuclear magnetic resonance (NMR) spectroscopy modalities for the evaluation of cerebral metabolism

### 3.1 Utility of NMR spectroscopy

NMR spectroscopy is well established as a powerful analytical technique for determining the structures of organic molecules. Labelling molecules, e.g. with ^13^C, enables their passage to be tracked along biochemical pathways, revealing the positions of the labelled atoms within metabolite molecules contained in biological samples. An example of ^13^C-labelled positions within molecules along metabolic pathways is illustrated in Fig. 3. Using cerebral microdialysis to deliver and collect ^13^C-labelled molecules in this context is a novel development (Gallagher et al., 2009).

Examples of ^13^C NMR spectra of brain microdialysates are illustrated in Fig. 4 (Gallagher et al., 2009). These illustrate that microdialysis perfusion with 2-^13^C acetate or 3-^13^C lactate produced ^13^C signals for glutamine C4, C3 and C2, indicating TCA cycle operation followed by conversion of glutamate to glutamine in TBI patients’ brains.

**Fig. 7:**
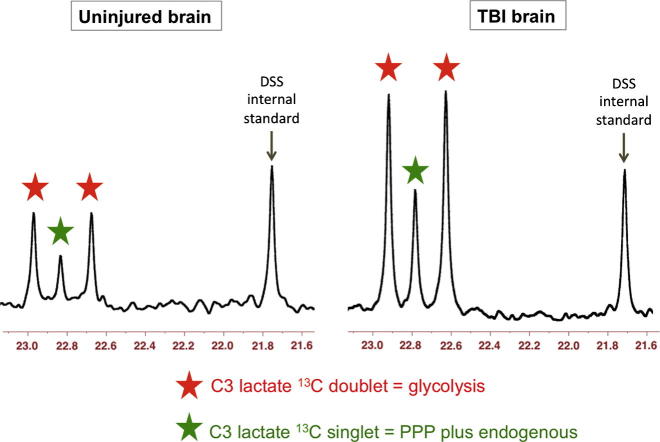
Examples of ^13^C NMR spectra of brain microdialysates from patients who received 1,2-^13^C_2_ glucose (4 mM) perfused via the microdialysis catheter (Jalloh et al., unpublished data). Uninjured brain is normal-appearing brain in a patient operated on for a benign tumour elsewhere in the brain. TBI brain is from a traumatic brain injury patient with diffuse injury. The part of the spectrum illustrated in each case is for the C3 carbon of lactate. Also present in this part of the spectrum is one of the signals due to the internal standard DSS (2,2-dimethyl-2-silapentane-5-sulfonate sodium salt). The remainder of the spectrum, including the main DSS signal (at 0 ppm) is not shown. The C3 doublet indicated by red stars represents lactate doubly labelled with ^13^C, produced by glycolysis; the C3 signal for ^13^C is split into 2 peaks by coupling to ^13^C also present at the neighbouring C2 position within the same molecule. The C3 singlet indicated by green stars represents lactate singly labelled with ^13^C, produced via the PPP; the singlet also contains a contribution from endogenous lactate due to the natural abundance background of ^13^C (1.1% of all carbon atoms).

In our current ongoing study, microdialysis perfusion with 1,2-^13^C_2_ glucose has resulted in ^13^C signals clearly visible as doublets for the C3 (and C2) of lactate, indicating glycolysis as a major pathway in TBI patients’ brains as expected. Small enrichment above natural abundance ^13^C was measurable in the lactate C3 singlet representing PPP production of lactate in brain. The PPP pathway is shown schematically in Fig. 5. Examples of the lactate C3 doublet and singlet pattern in ^13^C spectra of patients’ brain microdialysates are shown in Fig. 7 (Jalloh et al., unpublished data). No lactate C3 singlet enrichment was detectable in muscle above natural abundance, though glycolysis-derived lactate (C3 doublet) was seen.

Our novel combination of ^13^C-labelled microdialysis with analysis by high-resolution ^13^C NMR was the first direct demonstration in humans that the brain can utilise lactate via the TCA cycle (Gallagher et al., 2009), supporting the ANLS hypothesis (Pellerin and Magistretti 1994) (see Section 1.3). In our current study the ^13^C labelling patterns suggest that in TBI patients’ brains lactate production is mainly via glycolysis while PPP production of lactate is minor (see above). Further analyses of samples and data are ongoing in this study, which is the first direct comparison of glycolysis and PPP in human brain by ^13^C-labelled cerebral microdialysis. The technique will be extended to other substrates to further elucidate brain chemistry.

### 3.2 Ex vivo NMR of brain tissue extracts

In conventional studies of cerebral metabolism without microdialysis, ^13^C-labelled substrates are given intravenously to animals and then after appropriate time intervals the brains are removed and extracted (e.g. with perchloric acid) and NMR spectroscopy of the extracts (liquid solutions) is performed to determine ^13^C labelling in the products, either by using direct ^13^C-observe NMR e.g. (Bartnik et al., 2007; Bartnik et al., 2005), or indirect ^1^H-observe [^13^C-edited] NMR techniques such as spin-echo difference e.g. (Tyson et al., 2003). Analysis of brain tissue extracts *ex vivo* can give a global whole-brain picture of metabolism, or alternatively dissection of the brain prior to extraction can give region-specific information. Brain tissue extracts encompass both intra- and extra-cellular molecules, but are not available from humans unless they are undergoing surgery that excises portions of brain tissue (e.g. tumours, severe epilepsy), and cannot give a time-course within-subject; moreover in pre-clinical studies the animals have to be sacrificed.

NMR analysis can be performed directly on “solid” samples e.g. excised tissues (without having to extract the molecules), by high-resolution magic angle spinning NMR spectroscopy (HR-MAS NMR). By means of spinning samples at the “magic angle” (54.7° between the axis of rotation and the magnetic field), line-broadening processes such as dipole–dipole couplings, chemical shift anisotropy and magnetic susceptibility changes are significantly reduced for solid samples (Andrew et al., 1959). The tissue sample is placed in a small container termed a rotor (e.g. made of zirconium, 50 μl volume, 4 mm diameter), and is spun (e.g. at 5000 Hz) at a 54.7° angle inside the NMR magnet. This has been applied to characterise cells e.g. neuronal and glial cells grown *in vitro* studied by ^1^H HR-MAS NMR (Griffin et al., 2002), surgically excised tissue e.g. a ^1^H HR-MAS NMR study of human glioblastoma tissue (Piccirillo et al., 2012), and in ^13^C-labelling studies, e.g. a ^13^C HR-MAS NMR study of brain tissue (21–34 mg samples) from rats given intravenous 1,6-^13^C_2_ glucose (Risa et al., 2009).

### 3.3 NMR modalities: direct vs. indirect observation of ^13^C

NMR spectroscopy for measuring ^13^C-labelling can be either direct ^13^C-observe, or indirect ^1^H-observe [^13^C-edited]. An example of the latter is spin-echo difference spectroscopy e.g. (Tyson et al., 2003). The latter technique generates (a) a ^1^H NMR spectrum showing positive signals (upward-pointing peaks) from all protons, regardless of whether they are attached to ^12^C or ^13^C atoms, (b) a ^1^H spectrum in which protons attached to ^12^C give positive peaks but protons attached to ^13^C give inverted (negative, downward-pointing) peaks, and (c) a ^1^H difference spectrum, consisting of (a) minus (b) so that the peaks from protons attached to ^12^C are subtracted out and the peaks for protons attached to ^13^C appear positive.

While the indirect ^1^H-observe [^13^C-edited] spin-echo difference modality is more sensitive than the direct ^13^C-observe, the indirect modality is more complex to perform and interpret (see Section 3.4). An advantage of the spin-echo difference modality is that it gives information on fractional enrichment, i.e. ^13^C/(^12^C + ^13^C), by comparing the difference spectrum (c), which reveals protons attached to ^13^C, with the “all protons” spectrum (a) (see above). However, the ^13^C spectrum itself is unseen and there is no information on carbons without attached protons (e.g. carboxylate or ketone carbon).

For direct ^13^C-observe NMR, enrichments are determined by quantifying the ^13^C signal (by integration, i.e. measuring the peak areas, with reference to standards) and quantifying the “total” (labelled plus unlabelled) for the species of interest, either using direct ^1^H-observe NMR (integrating the signal with reference to standards), or by another analytical method such as HPLC or enzymatic colorimetric assays. To facilitate integrating NMR signals, peak fitting is available in NMR software packages (e.g. TopSpin and Chenomx). Reference standards consist of (a) an internal standard water-soluble reference substance, e.g. DSS (2,2-dimethyl-2-silapentane-5-sulfonate sodium salt) which provides a chemical shift reference point at zero ppm (*x-*axis of the spectrum) and acts as a reference for the signal response (*y*-axis) as it is added at a known, set concentration into every sample, and (b) an external standard calibration curve consisting of the molecular species of interest at an appropriate range of concentrations, plus DSS at the same fixed concentration as in (a). For direct ^13^C-observe NMR, the integrated signal intensity per ^13^C atom, i.e. the response factor, is not necessarily uniform (due to a natural effect termed Nuclear Overhauser Enhancement and other spin relaxation processes), so for each carbon position within each molecular species of interest, calibration has to be carried out.

The NMR modalities of direct ^13^C-observe, direct ^1^H-observe, and indirect ^1^H-observe [^13^C-edited] spin-echo difference each produce “one-dimensional” (1-D) spectra with signal response (*y*-axis) plotted vs. chemical shift (*x*-axis). Another NMR modality that is recently becoming applied to study metabolism is “two-dimensional” (2-D) NMR. For ^1^H-^13^C 2-D NMR, the analysis uses a ^1^H-optimised probe for acquisition with ^13^C-decoupling, and results are typically presented as a rectangular plot with a ^1^H-spectrum displayed along one axis (*x* or *y*), a ^13^C-spectrum along the second axis (*y* or *x*), and contours of the cross-peaks representing ^1^H–^13^C coupling in the “body” of the rectangle. Joining up the cross-peaks to the *x-* and *y-*axes establishes which carbons are connected to which protons. One type of ^1^H–^13^C 2-D spectroscopy is HSQC (heteronuclear single quantum coherence). This combines the high sensitivity of ^1^H spectroscopy with the high information content of ^1^H and ^13^C and their connectivity. It is more powerful than 1-D techniques for yielding unambiguous structural / identification information, largely overcoming the problems of unresolved signals that in some cases affect 1-D spectra (e.g. due to near-coincidences in chemical shift, and/or overlapping signals). ^1^H–^13^C HSQC was one of the techniques used to analyse the products of intravenous U-^13^C glucose in lung cancer patients, in a stable-isotope resolved metabolomics study (Fan et al., 2009). We have begun to use 2-D ^1^H–^13^C HSQC in selected cases, for additional verification of labelled products of ^13^C-labelled microdialysis, after routine 1-D ^13^C spectra and 1-D ^1^H spectra. We are also further interrogating the ^1^H spectra of the microdialysates by 2-D ^1^H–^1^H DQF-COSY (double quantum filtered correlation spectroscopy).

### 3.4 *In vivo* magnetic resonance spectroscopy

A ^13^C measurement technique applicable to both animals and humans is *in vivo* magnetic resonance spectroscopy (MRS). Though the description “nuclear” is generally not applied, the fundamental phenomenon is the same as in NMR. MRS is used in conjunction with MRI to measure the levels of metabolites *in situ* in chosen locations within body tissues, including regions of interest in the brain and is applied clinically to produce a “signature” which can assist diagnosis of certain disorders, e.g. tumours.

Microdialysis and MRS are complementary techniques, microdialysis focussing on the extracellular space, with MRS measuring combined extra- and intra-cellular metabolism. In addition, unlike microdialysis, which can only report on metabolic processes in the vicinity of the catheter, localised MRS can interrogate metabolism in multiple brain regions (using either natural abundance ^13^C MRS, or systemically administered ^13^C-labelled substrate). MRS could therefore allow comparison of metabolism in different tissue compartments (e.g. perilesional vs. normal appearing tissue). Comparison of extracellular chemistry in two sites within the same patient’s brain necessitates inserting a microdialysis catheter in each location, which is sometimes performed in clinical research. MRS can provide scope for non-invasive exploration of regional chemistry differences in brain.

Routine MRS on clinical MRI scanners usually measures ^1^H spectra in unlabelled brains. However, in research studies, direct *in vivo* MRS observation of ^13^C in brain has been performed with 1-^13^C glucose intravenous infusion in healthy human volunteers (Gruetter et al., 1994) and in patients (Moreno et al., 2001). These ^13^C-labelling studies have enabled glucose metabolism to be tracked to formation of glutamate and glutamine and in some cases TCA cycle rates have been estimated from the data e.g. (Mason et al., 1995). Also, 2-^13^C acetate intravenous infusions in healthy volunteers have enabled measurement, by direct ^13^C *in vivo* MRS, of astroglial metabolism to glutamate and glutamine (Lebon et al., 2002). There is much future scope for expanding the use of *in vivo* MRS in clinical studies utilising ^13^C labelling.

An alternative, indirect *in vivo* MRS method of detecting ^13^C labelling in brain utilises ^1^H-observe [^13^C-edited] spectroscopy, often abbreviated as POCE (proton observe, carbon edited) (Chen et al., 2001). It is more sensitive than directly-observed ^13^C MRS, since proton (^1^H) is inherently a more readily detectable nucleus than ^13^C (de Graaf et al., 2011). However design of the pulse sequences for acquisition of ^1^H-observe [^13^C-edited] spectra, and the interpretation of the data, is more complex. Also it has less scope as a screening technique - it works well for certain moieties, which need to be decided beforehand. The resultant spectra can be quite complex with incompletely resolved peaks (due to couplings) and overlapping signals within the narrow ppm scale of the proton (^1^H) spectrum, necessitating deconvolution by means of computation of modelled/fitted signals. An advantage of the ^1^H-observe [^13^C-edited] approach is that it directly gives ^13^C fractional enrichment values, i.e. the total (^12^C + ^13^C) and the ^13^C fraction of a given metabolite, analogous to the spin-echo difference NMR done in solution on animal brain tissue extracts *ex vivo*, e.g. (Tyson et al., 2003).

Another *in vivo* modality that has grown over the past decade is hyperpolarized ^13^C MRI/MRS (Brindle et al., 2011). While detailed discussion is outside the scope of the present article, some key examples can be mentioned. Until very recently, the technique was limited to pre-clinical studies, for practical reasons. Hyperpolarized 1-^13^C glutamate has been used to study tumour metabolism in cell cultures *in vitro* and in tumours *in vivo* in mice (Gallagher et al., 2011). Hyperpolarized 1-^13^C pyruvate and 2-^13^C pyruvate were used to study mitochondrial metabolism *in vivo* in normal rats treated with a putative anticancer agent, dichloroacetate that stimulates pyruvate dehydrogenase (Hu et al., 2012). Very recently, the first-in-man *in vivo* hyperpolarized ^13^C study was achieved, in prostate cancer patients, using 1-^13^C pyruvate (Nelson et al., 2013). Hyperpolarization, which results in a large but short-lived increase in signal-to-noise ratio, works best on ^13^C atoms of carboxylic acid or carbonyl functional groups (e.g. C1 or C2 of pyruvate). Because of the transient effect, the technique is best suited to study fast steps (e.g. pyruvate conversion to lactate, effected by lactate dehydrogenase) rather than long sequences of multiple steps, in metabolic pathways. Hyperpolarization appears probably too short-lived for the microdialysis timescale. However the ^13^C-labelled species could, in principle, be analysed conventionally after the hyperpolarization has subsided, so microdialysis could be used in a complementary role in hyperpolarization studies.

## 4 Strengths and limitations of ^13^C-labelled microdialysis

### 4.1 Microdialysis and NMR

Microdialysis is a continuous-flow technique, which is advantageous for monitoring patients, though its inherently low flow rates place demands on analysis, due to small samples. For our ^13^C studies we use 0.3 μl/min, the same as for neurocritical care microdialysis monitoring in non-labelled cases (Timofeev et al., 2011). We have not adopted higher microdialysis flow rates (e.g. 2 μl/min), as although these would produce larger volumes the samples would be more dilute, and high flow rates might theoretically perturb the tissue biochemistry. Having a low flow rate (0.3 μl/min) means better equilibration between the solution in the catheter and the brain extracellular fluid. To have sufficient material for ^13^C-NMR necessitates pooling the contents of multiple microdialysate collection vials. At our standard flow rate of 0.3 μl/min, we have pooled 24 h of microdialysate collection vials per NMR tube (Gallagher et al., 2009). Our NMR spectrometer is a Bruker Avance 500 MHz (11.7 Tesla magnet), thus ^13^C spectra are at 125.75 MHz. With the narrowest conventional NMR sample tubes (3 mm) it is possible to analyse down to a half-day of microdialysate. Inside the magnet, the NMR sample tube sits within a device termed a probe. Ours is a cryoprobe, which possesses greater sensitivity than non-cryo probe technology. In a cryoprobe, the sample is kept at ambient (room) temperature while cold helium gas is used cool the radio-frequency transmitter and receiver coils (ca. 16 K) and preamplifier (77 K), for improved signal-to-noise ratio. Cryoprobe technology enables acquisition of ^13^C spectra of pooled microdialysates within a reasonable timeframe. For example, our 24 h-pooled microdialysates were analysed on NMR for 4096 scans with a 3s delay between each scan (Gallagher et al., 2009), i.e. an analysis time of approximately 3.4 h. Without a cryoprobe the ^13^C spectrum would necessitate a day or more of NMR time to acquire, which is impractical. Recently, NMR micro-cryoprobes have become commercially available, for smaller samples (e.g. 30 μl for Bruker’s micro-cryoprobe), which would enable better time-resolution in measurements of metabolic ^13^C-labelling in future, making it theoretically possible to analyse 2 h pools of microdialysates.

NMR strengths are that it identifies the position of the labelled atom within the metabolite molecule, very little sample manipulation is required, it is non-destructive, and it can be used for screening, not requiring prior selection of analytes to seek. ^13^C-labelled microdialysis can be used safely for severe TBI patients without interrupting their standard medical care. ^13^C is a stable isotope and not radioactive. ^13^C-labelled microdialysis micro-doses a small region of the brain directly, avoiding any issues with import across the blood–brain barrier. It also avoids any possible adverse effects associated with systemic dosing, which (in humans) would necessitate intravenous administration of large, expensive amounts of ^13^C substrate that would be potentially complicated by metabolism by the liver and/or other non-cerebral tissues. With ^13^C-labelled cerebral microdialysis it is only necessary to purchase small amounts (e.g. 1 or 2 g) of the ^13^C-labelled substrate to make up the formulation in CNS perfusion fluid, sufficient for multiple patients, as amounts and volumes delivered are so small.

Like all microdialysis methodology, ^13^C-labelled microdialysis is an invasive technique. Cerebral microdialysis cannot be applied to healthy volunteers and is limited to patients monitored in neurocritical care for severe brain injury (e.g. head injury and certain types of stroke), and patients undergoing surgery (e.g. severe epilepsy, tumour resection or biopsy). There is thus a problem in finding normal brain to act as a control. The best available approximation is normal-appearing brain in patients undergoing surgery for benign tumours in a different area of the brain.

Microdialysis is limited to the extracellular pool, so may miss detecting labelled species if they are predominantly intracellular. Moreover, microdialysis is a focal technique, limited to monitoring the immediate vicinity of the catheter (Section 2.2). Information from ^13^C-labelled microdialysis may in future be complemented by studies with *in vivo* MRS that addresses the whole tissue (combined intracellular and extracellular molecules) in selected region(s) of interest (Section 3.4).

### 4.2 Mass spectrometry for measuring ^13^C-labelling in microdialysates

Mass spectrometry is an alternative to NMR for measuring ^13^C-labelling in microdialysates. Gas chromatography–mass spectrometry (GC–MS) was used to analyse human adipose tissue microdialysates in healthy volunteers (Gustafsson et al., 2007). In that study, 1-^13^C glucose was infused via the microdialysis catheter and ^13^C labelling measured by GC–MS in the products lactate and glycerol recovered in the microdialysates. Apart from that study, and our study (Gallagher et al., 2009), we are unaware of any other published studies that used microdialysis to both deliver ^13^C-labelled substrates and collect the ^13^C-labelled products.

GC–MS was used to analyse ^13^C labelling in glutamate, in brain microdialysates from rats receiving intravenous 2,5-^13^C_2_ glucose, with and without a glutamate uptake inhibitor (Kondrat et al., 2002). Another study, in rats that received an intravenous infusion sequence of 2,5-^13^C_2_ glucose, ^12^C glucose, and ^15^NH_4_Ac (ammonium acetate) involved *in vivo* ^13^C MRS, *in vivo* microdialysis, ^13^C NMR for *ex vivo* analysis of brain tissue extracts, and GC–MS for analysis of microdialysates (Kanamori et al., 2002). An advantage of mass spectrometry is that it can be used for any stable isotope label, even those not amenable to NMR analysis. For GC-MS, a disadvantage is that processing and derivatisation steps are usually required to prepare the samples for analysis, to render the molecule(s) of interest sufficiently volatile. Other “hyphenated” mass spectrometry techniques not requiring derivatisation, e.g. liquid chromatography (LC–MS, or LC–MS/MS), may be useful in future for labelled microdialysis studies.

Mass spectrometry is more sensitive than NMR for the amount of sample needed, and mass spectrometry gives a direct measure of percentage enrichment of ^13^C within the molecule. However, mass spectrometry usually does not give information about the position of the ^13^C label within the molecule, unless suitable fragment ions are generated. Tandem mass spectrometry (e.g. GC–MS/MS or LC–MS/MS) offers controlled fragmentation which, depending on the species in question, may yield positional information on labelling patterns (Antoniewicz 2013). This methodology has begun to be applied to metabolic flux analysis, and with future refinement may gain wider use in this context (Antoniewicz 2013). Position of the label, determined by NMR is the “gold standard” for verifying the biosynthetic pathway.

### 4.3 Limitations due to isotope background

An ultimate limitation of ^13^C labelling is the naturally occurring background (termed natural abundance) since 1.1% of all carbons are ^13^C. This limits the sensitivity of detecting low amounts of exogenously supplied label, especially when there is a substantial concentration of the endogenous molecule naturally present. This limitation is particularly marked when the molecule of interest is singly labelled with ^13^C, though can largely be overcome by using double labelling. Having two ^13^C atoms adjacent to each other in the molecule of interest produces a “signature” of doublets in the ^13^C NMR spectrum. The likelihood of this occurring naturally is 0.012%. However, if a biosynthetic pathway results in loss of one of the two ^13^C labelled atoms (as in the PPP, with 1,2-^13^C_2_ glucose substrate), which would then give a singly-labelled product, there will be a doublet background of 1.1% in the product, due to the one remaining “label” ^13^C atom being next to a natural background ^13^C atom thereby creating a “pseudo-doubly labelled” situation. Given that glycolysis is the major pathway converting 1,2-^13^C_2_ glucose into 2,3-^13^C_2_ lactate thereby showing lactate C3 (and C2) doublets that are typically strong signals, the contribution of pseudo-doubly labelled lactate to the observed C3 doublet signal may be relatively minor. However, “reimbursing” the C3 singlet PPP-derived lactate signal by adding on the pseudo-doubly labelled component of the C3 doublet may increase the estimation of the PPP.

The natural abundance background of ^13^C also affects mass spectrometry. The latter is inherently more sensitive than NMR, so mass spectrometry can be done with smaller amounts of sample. However, mass spectrometry has the disadvantage (mentioned earlier) that it does not usually distinguish the intra-molecular positions of enrichment and in most cases mass spectrometry just determines the ion intensity ratios of the molecular ions. Thus ^13^C enrichments in all positions (carbon atoms) within the molecule of interest are combined together on mass spectrometry so that the higher molecular weight of the molecule, the higher the background signal. So, for example, on mass spectrometry a molecule with 3 carbons will have a 3.3% (3  × 1.1%) background contribution to singly labelled molecular ion (M+1, where M is the molecular weight of the unlabelled molecule) due to natural abundance ^13^C. Furthermore, derivatisation procedures employed for GC–MS analysis also contribute to background since the derivatised form of the molecule contains extra atoms. In contrast, NMR signals are specific to each individual carbon position so the background stays at 1.1%. On mass spectrometry, the background at singly labelled molecular ion (M+1) is further increased by the contributions of the naturally-occurring stable isotopes ^2^H, ^17^O and ^15^N with natural abundances 0.015%, 0.039% and 0.366% respectively, each multiplied by the number of H, O and N atoms respectively per molecule then added onto the ^13^C background on mass spectrometry. These non-^13^C isotopes do not contribute to the background on the ^13^C NMR spectrum. For doubly-labelled molecular ion (M+2) the natural abundance of ^18^O (0.201%), multiplied by the number of O atoms per molecule, contributes a background on mass spectrometry. Background contributions to M+1 and M+2 from isotopes of silicon will affect silyl-derivatised molecules on GC–MS, e.g. due to ^29^Si (4.67%) and ^30^Si (3.1%). Adoption of sufficiently high-resolution mass spectrometry may be able to discriminate the “isobars” component of background in the molecular ion due to non-^13^C isotopes, but the component of background due to natural abundance ^13^C would of course remain present.

A further consideration of natural abundance background ^13^C is that each step of a biosynthetic pathway may introduce progressive dilution with endogenous molecules, depending on the pathway and pool size, and any competing, branching, or following-on pathways that would “lose” molecules. Also, self-dilution occurs if a molecule labelled at one end breaks into two as part of a pathway – e.g. in glycolysis, one 1-^13^C glucose molecule produces one 3-^13^C pyruvate and one unlabelled pyruvate. For microdialysates, some labelled species may be missed if they are predominantly intracellular (e.g. glutamate). Such considerations may help explain why ^13^C-labelling was negligible in glutamate or glutamine in cerebral microdialysates resulting from perfusion of TBI patients’ brains with 1-^13^C glucose (2 mM, 99% enriched) via the microdialysis catheter (Gallagher et al., 2009). Dilution and losses can be partly offset by starting with a high concentration of labelled substrate, at high% enrichment (e.g. 99%), although increasing the absolute concentration (regardless of label) risks perturbing the biochemistry. ^13^C label might theoretically affect some reaction rates, but the relative mass difference (^13^C vs. ^12^C) is so small that any effect is usually deemed negligible.

## 5 Conclusions and future directions

Using microdialysis to both deliver and collect ^13^C-labelled molecules to and from the brain extracellular fluid enables site-specific information from patients in a local micro-dosing regimen. For analysing the microdialysates, NMR spectroscopy is the “gold standard” powerful tool for tracking ^13^C labelling, revealing not only which molecular species are labelled, but also precisely which intra-molecular position(s) the label is in, enabling the biosynthetic pathway(s) to be deduced. ^13^C NMR is ideally suited to energy metabolism studies, where the levels of molecules of interest are often relatively large, of the millimol/L order, though in principle NMR is applicable to other biosynthetic pathways. Future adoption of NMR micro-cryoprobe technology would permit smaller samples, enabling better time-resolution for the microdialysates by reducing requirements for pooling. Further downscaling of the microdialysate sample size requirements is possible with analysis by mass spectrometry, though at the expense of at least some of the detailed information on precise intra-molecular position of label. Even so, mass spectrometry has the potential to track labelling in smaller time-resolved samples and into low-concentration intermediates/metabolites that are too low for NMR. Overall, ^13^C-labelled microdialysis methodology, with analysis by high-resolution NMR and mass spectrometry, widens the scope of microdialysis for use in clinical studies of energy metabolism, drugs and other therapeutic interventions.
